# Tailoring the surface area and the acid–base properties of ZrO_2_ for biodiesel production from *Nannochloropsis* sp.

**DOI:** 10.1038/s41598-019-52771-9

**Published:** 2019-11-07

**Authors:** Nurul Jannah Abd Rahman, Anita Ramli, Khairulazhar Jumbri, Yoshimitsu Uemura

**Affiliations:** 1 Fundamental and Applied Sciences Department, Universiti Teknologi PETRONAS, 32610 Seri Iskandar, Perak, Malaysia; 2 Centre for Biofuel and Biochemical Research, Universiti Teknologi PETRONAS, 32610 Seri Iskandar, Perak, Malaysia

**Keywords:** Chemistry, Energy science and technology

## Abstract

Bifunctional heterogeneous catalysts have a great potential to overcome the shortcomings of homogeneous and enzymatic catalysts and simplify the biodiesel production processes using low-grade, high-free-fatty-acid feedstock. In this study, we developed ZrO_2_-based bifunctional heterogeneous catalysts for simultaneous esterification and transesterification of microalgae to biodiesel. To avoid the disadvantage of the low surface area of ZrO_2_, the catalysts were prepared via a surfactant-assisted sol-gel method, followed by hydrothermal treatments. The response surface methodology central composite design was employed to investigate various factors, like the surfactant/Zr molar ratio, pH, aging time, and temperature on the ZrO_2_ surface area. The data were statistically analyzed to predict the optimal combination of factors, and further experiments were conducted for verification. Bi_2_O_3_ was supported on ZrO_2_ via the incipient wetness impregnation method. The catalysts were characterized by a variety of techniques, which disclosed that the surfactant-assisted ZrO_2_ nanoparticles possess higher surface area, better acid–base properties, and well-formed pore structures than bare ZrO_2_. The highest yield of fatty acid methyl esters (73.21%) was achieved using Bi_2_O_3_/ZrO_2(CTAB)_, and the catalytic activity of the developed catalysts was linearly correlated with the total densities of the acidic and basic sites. The mechanism of the simultaneous reactions was also discussed.

## Introduction

Biodiesel is an attractive alternative source of energy owing to its renewability, biodegradability, sustainability, and non-toxicity^1^. It is produced by transesterification of vegetable oil or animal fat with short-chain alcohols in the presence of suitable chemical catalysts (homogeneous/heterogeneous) or enzymatic biocatalysts^2–4^. Today, there has been growing research interest in using microalgae as biodiesel feedstock because of its rapid growth rate, high photosynthetic efficiency, and high oil contents, as well as the minimum space needed for cultivation^5,6^. Industrially, conventional homogeneous catalysts are used in the transesterification process for the production of biodiesel^2^. However, the catalysts require extensive washing and purification steps, and they cause undesired saponification when dealing with high-free-fatty-acid (FFA) content feedstock^7^. The enzymatic transesterification of lipases is commonly associated with high production cost and fast deactivation at severe reaction conditions that limit its application at an industrial scale^8^. An alternative method to overcome these challenges is the utilization of heterogeneous catalysts.

Numerous studies have been reported on heterogeneous catalysis for biodiesel production. The most common key features of efficient and active heterogeneous transesterification catalysts are high surface area^9,10^, adequate acidic^11–13^ and basic^14–17^ densities, good crystallinity^14^, and -well-formed pore structure^10,12,13^. Recent developments in this field have led to renewed interest in bifunctional acid–base heterogeneous catalysts for simultaneous esterification and transesterification of low-grade high-FFA model feedstock^7,18,19^, such as microalgae lipid. Heterogeneous acidic catalysts are commonly used for the esterification step as the reaction is less affected by the presence of water and FFA. Instead, heterogeneous basic catalysts are employed in the second transesterification step because they are more active than acidic catalysts, which require shorter reaction time and lower reaction temperature^20^. Some studies have reported the use of bifunctional heterogeneous catalyst for biodiesel production^18,19,21–23^. However, reports on their application using microalgae lipid as the biodiesel feedstock are still limited.

Zirconium dioxide (zirconia, ZrO_2_) is a well-known heterogeneous catalyst and catalyst support that exhibits unique characteristic of amphoteric nature which indicates its remarkable potential to perform simultaneous esterification–transesterification reactions of high-FFA feedstock to biodiesel^19^. ZrO_2_ has a high boiling point, high melting point, good thermal stability, and good corrosion resistance, making it an excellent heterogeneous catalyst even under harsh reaction conditions^24,25^. As a catalyst support, ZrO_2_ exhibits better chemical properties and higher stability than the traditional catalyst supports of γ-alumina and silica^26^. Among the common techniques of synthesizing ZrO_2_ are sol-gel^27,28^, precipitation^24,29^, microwave-assisted^30^, ultrasound-assisted^31,32^, and emulsion^33^ methods. However, one of the biggest challenges that has limited its performance in practical applications so far^24^ is the development of a suitable synthetic route of ZrO_2_ with a high surface area, adequate acid–base properties, good crystalline structure, and well-developed porosity for the aforementioned purpose.

Several attempts have been adopted to improve the surface area of heterogeneous catalysts through the synthesis of nanoscale materials by surfactant-assisted methodologies^34,35^. The surfactant plays a decisive role in tailoring the properties of the heterogeneous catalysts, including its shape and size, which, in turn, depend on the nature of the surfactant, such as the length of the hydrophobic tail and the ions (cationic, anionic, or non-ionic) ^36^. Previous studies suggested the use of surfactant in the sol-gel technique because of its homogeneity and ability to control the surface area, the pore volume, and the pore size distribution of the catalysts^31,37^. The synthesis of surfactant-assisted ZrO_2_ catalyst is governed by a variety of parameters, including the surfactant type and the synthetic conditions that affect its overall quality. For instance, Eltejaei *et al*. used poly(ethylene glycol)-*block*-poly(propylene glycol)-*block*-poly(ethylene glycol) (PEG–PPG–PEG) as a non-ionic surfactant in the synthesis of tetragonal ZrO_2_, employing the precipitation method at basic Ph^34^. Alteration of the pH from 10 to 11 resulted in high surface area ZrO_2_ due to the increase in surface charge and nucleation that occurs at high pH values. In another study, Zhang *et al*. synthesized nano-sized tetragonal ZrO_2_ via hydrothermal treatment using cetrimonium bromide (CTAB) as the cationic surfactant. Hydrothermal energy, a non-conventional energy source for the synthesis of nanoparticles, prevents particle agglomeration and allows for uniform grain size and regular morphology^38^.

In the present study, we developed an effective ZrO_2_-based bifunctional heterogeneous catalyst for simultaneous esterification–transesterification of microalgae lipid to biodiesel. The effect of several process parameters on the surface area of ZrO_2_ prepared by a surfactant-assisted sol-gel method followed by a hydrothermal treatment using non-ionic and cationic surfactants under basic conditions was investigated. The optimization of the process parameters was achieved using response surface methodology central composite design (RSMCCD). Mathematical models were developed and validated to predict the maximum surface area of ZrO_2_. The acidic and basic properties of ZrO_2_ were tailored after modification with bismuth oxide (Bi_2_O_3_) via incipient wetness impregnation method. The synthesized catalysts were found to be active towards the conversion of microalgae lipid to biodiesel.

## Results and Discussion

### Effect of the type of the surfactants and the reaction parameters on the surface area of ZrO_2_ and optimization study

The maximum surface area of ZrO_2_ was achieved using poly(ethylene oxide)-block-poly(propylene oxide)-block-poly(ethylene oxide) (Pluronic P123) and cetrimonium bromide (CTAB) as surfactants, yielding the ZrO_2(P123)_ and ZrO_2(CTAB)_ catalysts, respectively. The optimization of the process parameters was conducted by employing RSMCCD, which maintained the experimental conditions within the desired range of independent parameters. According to the literature, the most important parameters affecting the surface area of ZrO_2_ are the surfactant/Zr ratio (A), pH (B), aging time (C), and temperature (D) ^39,40,41^. The specific values of the independent parameters used in this study, along with the surface area obtained for ZrO_2(P123)_ and ZrO_2(CTAB)_ are cited in Supplementary Tables S1 and S2, respectively. Specifically, among the 30 experimental RSMCCD runs, ZrO_2(P123)_ displayed a maximum surface area of 79 m^2^/g (Run 21), whereas ZrO_2(CTAB)_ exhibited a maximum surface area of 295 m^2^/g (Run 20).

The relationship between the independent parameters and the surface area obtained using the analysis of variance (ANOVA) test for ZrO_2(P123)_ and ZrO_2(CTAB)_ are summarized in Supplementary Tables S3 and S4, respectively. By fitting the data to various polynomial models, the ANOVA result shows that both ZrO_2(P123)_ and ZrO_2(CTAB)_ were suitably fitted to reduced cubic models. The obtained P-values (< 0.05) indicated that the suggested model terms have a significant effect on the response^42^. In particular, for ZrO_2(P123)_ (R1), the significant terms were A, B, C, D, CD, B^2^, C^2^, D^2^, ACD, and A^2^B, and for ZrO_2(CTAB)_ (R2), the significant terms were C, BC, CD, A^2^, B^2^, C^2^, D^2^, BCD, A^2^C, and A^2^D. Herein, the combined effect of aging time and temperature (CD) was one of the most significant terms toward the improvement of the ZrO_2_ surface area. Thus, this observation highlighted the importance of sufficient aging time for the effective distribution of Zr–OH and Zr–O–Zr in order to form a stable network gel. Moreover, it was proven that a suitable hydrothermal temperature leads to the development of internal pressure, increases in the motion velocity of the surfactants, and prevents the agglomeration of the Zr nanoparticles^38^.

The high coefficient of determination (R^2^) obtained indicated the goodness of fit of the two generated models. Furthermore, the lack of fit values for both models were not significant which is the desirable result. Both R^2^ and adjusted R^2^ values were close to unity, indicating the accuracy of the models. The low values of the coefficient of variation (CV%) for both models indicated the good precision and reliability of the experiments. The correlation between the predicted and actual surface areas of ZrO_2(P123)_ and ZrO_2(CTAB)_ are shown respectively in Supplementary Fig. S1 (a,b). The relationship between the predicted and actual values for both models was approximately linear, pointing out the reliability of the models developed to establish a correlation between the process parameters and the surface area. Accordingly, the final predicted surface areas of ZrO_2(P123)_ and ZrO_2(CTAB)_ were determined based on the given values of each factor, as shown in Eqs (1) and (2), respectively:

1 $$\begin{array}{rcl}R1 & = & 64.14+1.17A-2.51B-1.79C+2.74D-0.0119AB\\  &  & -\,0.0056AC+0.0344AD+0.2769BC-0.4531BD\\  &  & -\,3.18CD-0.5026{A}^{2}+1.88{B}^{2}+2.66{C}^{2}+1.55{D}^{2}\\  &  & +\,0.6956ACD+3.04{A}^{2}B\end{array}$$

2 $$\begin{array}{rcl}R2 & = & 274.42+2.23A-3.68B+25.83C+7.54D-1.23AB\\  &  & +\,2.48AC+3.33AD-8.25BC-5.90BD\\  &  & +\,47.18CD-52.12{A}^{2}-52.36{B}^{2}-39.59{C}^{2}-27.33{D}^{2}\\  &  & +\,2.76ACD-8.15BCD+22.65{A}^{2}C+38.17{A}^{2}D\end{array}$$

where, A is the surfactant/Zr molar ratio, B is the pH value, C is the aging time, and D is the temperature.

The optimum reaction parameters suggested by RSMCCD for the highest surface area of ZrO_2(P123)_ and ZrO_2(CTAB)_ are summarized in Supplementary Table S5. Specifically, the optimum surface area of ZrO_2(P123)_ was 79 m^2^/g using a surfactant/Zr molar ratio of 0.03, pH of 9.5, and aging time of 22 h at 110 °C. On the other hand, an optimum surface area of 295 m^2^/g was achieved for ZrO_2(CTAB)_ using a surfactant/Zr molar ratio of 0.89, pH of 9.8, and aging time of 39 h at 110 °C. The deviation (%) values were calculated according to the deviation between the predicted and experimental values^43^. The results obtained were satisfactory and reliable, with acceptable proximity.

### Catalyst characterization

**Figure 1:**
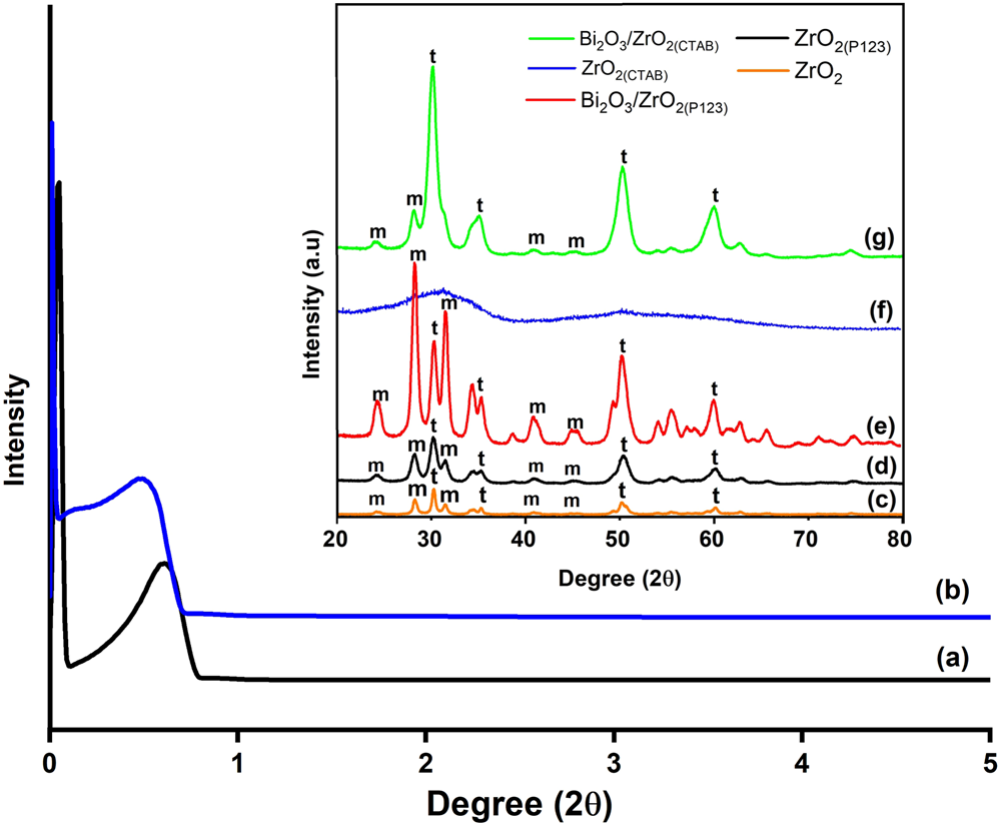
Low-angle XRD diffractograms of (**a**) ZrO_2(P123)_ and (**b**) ZrO_2(CTAB)_ and wide-angle XRD diffractograms (inset) of (**c**) bare ZrO_2_, (**d**) ZrO_2(P123)_, (**e**) Bi_2_O_3_/ZrO_2(P123)_, (**f**) ZrO_2(CTAB)_, and (**g**) Bi_2_O_3_/ZrO_2(CTAB)_ catalysts. *t* and *m* refer to the tetragonal and monoclinic ZrO_2_, respectively.

Figure 1(a–g) illustrate the low-angle and wide-angle X-ray diffraction (XRD) spectra of the synthesized catalysts. The presence of low-angle diffraction peaks at a 2θ range from 0.06° to 0.80° indicates that both ZrO_2(P123)_ and ZrO_2(CTAB)_ exhibited well-organized mesopore structures after calcination at 500 °C (Fig. 1a,b) ^44,45^. Figure 1c shows the wide-angle XRD diffractogram of bare ZrO_2_ with reflection peaks at 2θ = 30.2° (011), 35° (110), 50.3° (112), and 60.1° (121) that corresponded to tetragonal ZrO_2_ (t-ZrO_2_; ICDD: 98-015-7619). The remaining peaks at 2θ = 24.4° (110), 28.3° (11-1), 31.5° (111), 40.8° (102), and 45° (211) corresponded to monoclinic ZrO_2_ (m-ZrO_2_; ICDD: 98-006-8782). It was observed that the addition of Pluronic P123 (Fig. 1d) slightly increased the intensity of the peaks but did not shift the peaks position. The addition of a small amount of Bi_2_O_3_ on the surface of ZrO_2(P123)_ has significantly enhanced the crystalline structure of the final catalyst by forming sharp and highly intense peaks at the same 2θ values (Fig. 1e). On the other hand, the addition of CTAB (Fig. 1f) resulted in two broad and unremarkable peaks centered at 2θ = 30.7° and 50.6°, indicating an amorphous structure. The amorphous structure of ZrO_2(CTAB)_ was also crystallized to almost tetragonal phase after impregnation with Bi_2_O_3_ (Fig. 1g) due to its instability which allowed phase transitions^46^. Although the peaks of Bi_2_O_3_ could not be identified because of overlapping with the peaks of m-ZrO_2_, the cubic phase of Bi_2_O_3_ (ICDD: 98-000-2375) was present at 2θ = 27.2° (111), 31.5° (002), and 45.2° (022).

Table 1 shows the average crystallite sizes and compositions of the m-ZrO_2_ and t-ZrO_2_ forms of the catalysts. Bare ZrO_2_ possessed larger average crystallite sizes for both monoclinic and tetragonal phases compared to the Pluronic- and CTAB-assisted nanoparticles. During the synthesis of ZrO_2_, the surfactant served as a soft template to prevent agglomeration of nanoparticles through various repulsive and attractive forces that developed between the surfactant and the nanoparticles^36^. The average crystallite sizes of ZrO_2(P123)_ were found to be 11.5 and 10.3 nm for the monoclinic and tetragonal phases, respectively. The insertion of Bi_2_O_3_ into the ZrO_2(P123)_ framework increased the crystallite size of the catalyst because of the participation of Bi_2_O_3_ in the growth of the particles. Since ZrO_2(CTAB)_ was amorphous, no XRD data related to crystallite size were obtained. However, the crystallite sizes of m-ZrO_2_ and t-ZrO_2_ in Bi_2_O_3_/ZrO_2(CTAB)_ were determined at 10.7 and 9.3 nm, respectively. Rietveld quantitative analysis was applied as a powerful tool to quantify the crystalline components in the multiphase structures^47^. As outlined in Table 1, the volume fractions of the monoclinic and tetragonal phases of bare ZrO_2_ were similar. Initially, ZrO_2(P123)_ was predominantly in the tetragonal phase. However, loading of Bi_2_O_3_ on the ZrO_2(P123)_ surface transformed the tetragonal to the monoclinic phase. By contrast, Bi_2_O_3_/ZrO_2(CTAB)_ has a higher content of t-ZrO_2_ compared to m-ZrO_2_. In many reaction systems, t-ZrO_2_ has been reported to show high catalytic activity^48^ because of its low surface energy^49^ and its optimum geometrical arrangement that stabilizes a transition state complex between the reactants on the t-ZrO_2_ surface^50^.

**Table 1:** Average crystallite sizes and phase compositions of the m-ZrO_2_ and t-ZrO_2_ forms of the synthesized catalysts.

Catalyst	Crystallite size (nm)		Phase composition (vol %)	
	m-ZrO_2_	t-ZrO_2_	V_m_	V_t_
ZrO_2_	21.3	30.2	48.3	51.7
ZrO_2(P123)_	11.5	10.3	43.9	56.1
Bi_2_O_3_/ZrO_2(P123)_	14.5	12.3	77.9	22
ZrO_2(CTAB)_	—	—	—	—
Bi_2_O_3_/ZrO_2(CTAB)_	10.7	9.3	21.6	78.4

**Figure 2:**
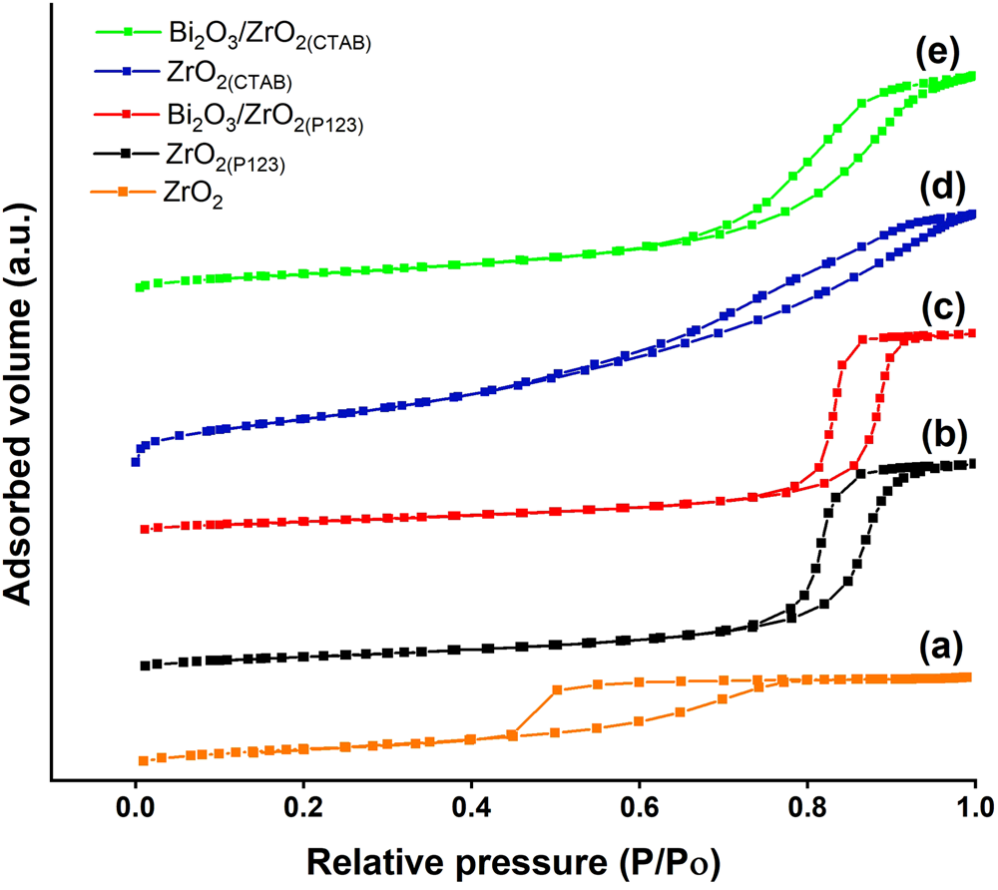
Nitrogen adsorption/desorption isotherms of (**a**) bare ZrO_2_, (**b**) ZrO_2(P123)_, (**c**) Bi_2_O_3_/ZrO_2(P123)_, (**d**) ZrO_2(CTAB)_, and (**e**) Bi_2_O_3_/ZrO_2(CTAB)_ catalysts.

**Figure 3:**
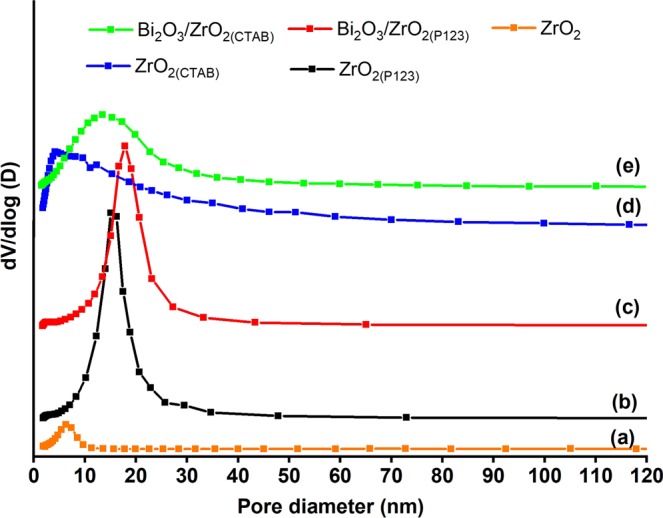
Pore distribution plots of (**a**) bare ZrO_2_, (**b**) ZrO_2(P123)_, (**c**) Bi_2_O_3_/ZrO_2(P123)_, (**d**) ZrO_2(CTAB)_, and (**e**) Bi_2_O_3_/ZrO_2(CTAB)_ catalysts.

Figure 2 shows the nitrogen adsorption/desorption isotherms of the catalysts. According to IUPAC classification, all of the catalysts exhibited a type IV isotherm with a hysteresis loop because of capillary condensation attributed to the well-developed mesoporous system^51^. The shape of the hysteresis loop contributed to the characteristic specific pore structures in the catalysts. According to the obtained results, bare ZrO_2_, ZrO_2(CTAB)_, and Bi_2_O_3_/ZrO_2(CTAB)_ resembled the H2 type, typical for inorganic oxides with ink-bottle-shaped mesopores^52^. ZrO_2(P123)_ and Bi_2_O_3_/ZrO_2(P123)_ exhibited a H1 type of hysteresis loop, implying the existence of a cylindrical pore geometry, spherical particles compacted in uniform arrangement, and a high degree of pore size uniformity^52,53^. The Brunauer–Emmett–Teller (BET) surface area, the total pore volume, and the average pore size are outlined in Table 2. Bare ZrO_2_ exhibited the lowest values of surface area and total pore volume. Regarding the surfactant-assisted nanoparticles, ZrO_2(CTAB)_ exhibited a significantly larger BET surface area and total pore volume than ZrO_2(P123)_. This is due to the higher CTAB/Zr molar ratio used in the synthesis of ZrO_2(CTAB)_ and the effect of the surfactant’s cationic nature. After mixing water with CTAB, the cationic charges of CTAB were released and induced repulsive forces between the Zr particles, which resulted in a high pore volume^54^. The higher pore volume along with smaller average pore size contributed to the formation of a higher total surface area. However, the total surface areas of ZrO_2(P123)_ and ZrO_2(CTAB)_ decreased by about 20% and 47%, respectively, after impregnation with Bi_2_O_3_ because of the pore filling effect. In addition, all of the catalysts exhibited a mesoporous structure with an average pore size between 5.6 and 13.98 m^2^/g. Figure 3 shows the pore size distribution plots using the Barrett, Joyner, and Halenda method. All of the catalysts exhibited unimodal pore size distribution plots, with ZrO_2(P123)_ and Bi_2_O_3_/ZrO_2(P123)_ showing a narrower pore size distribution compared to the other catalysts, indicating their high degree of pore size uniformity. After the impregnation with Bi_2_O_3_, an increase in the average pore size of Bi_2_O_3_/ZrO_2(P123)_ and Bi_2_O_3_/ZrO_2(CTAB)_ was observed because of the shifting of the pore size plots to the right of the larger pore size area^55^.

**Table 2:** BET surface area, total pore volume, and average pore size of the synthesized catalysts.

Catalyst	BET surface area (m^2^/g)	Total pore volume (cm^3^/g)	Average pore size (nm)
ZrO_2_	37	0.06	5.6
ZrO_2(P123)_	79	0.31	10.79
Bi_2_O_3_/ZrO_2(P123)_	63	0.26	13.98
ZrO_2(CTAB)_	295	0.58	5.69
Bi2O_3_/ZrO_2(CTAB)_	157	0.31	9.80

**Figure 4:**
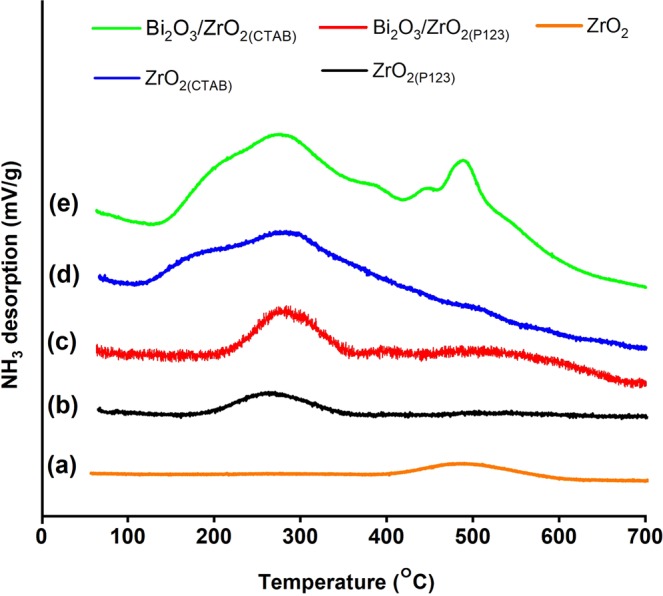
NH_3_-TPD profiles of (**a**) bare ZrO_2_, (**b**) ZrO_2(P123)_, (**c**) Bi_2_O_3_/ZrO_2(P123)_, (**d**) ZrO_2(CTAB)_, and (**e**) Bi_2_O_3_/ZrO_2(CTAB)_ catalysts.

**Figure 5:**
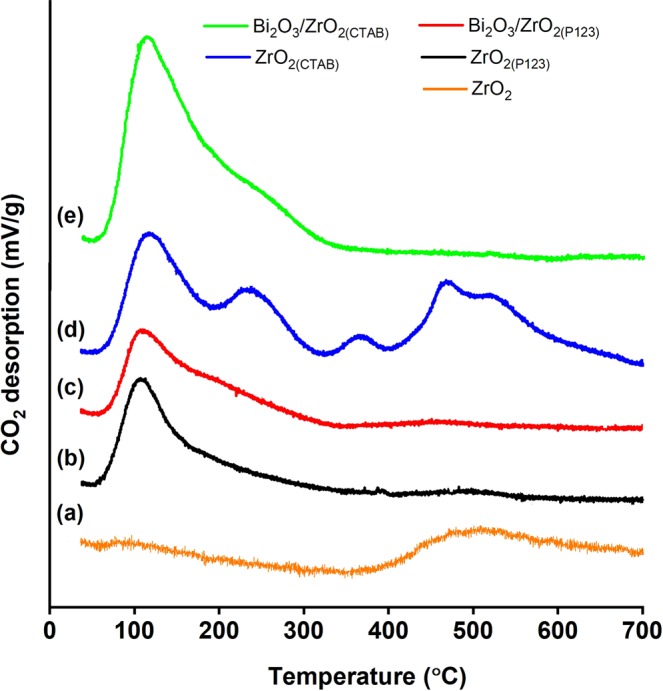
CO_2_-TPD profiles of (**a**) bare ZrO_2_, (**b**) ZrO_2(P123)_, (**c**) Bi_2_O_3_/ZrO_2(P123)_, (**d**) ZrO_2(CTAB)_, and (**e**) Bi_2_O_3_/ZrO_2(CTAB)_ catalysts.

The acid–base bifunctional properties of the catalysts were proven by the NH_3_-TPD and CO_2_-TPD profiles, as depicted in Figs 4 and 5, respectively. The total acidic/basic sites of the catalysts along with their density are summarized in Table 3. The number of total acidic/basic sites was calculated based on the intensity of the NH_3_/CO_2_ desorption peaks, and the density of each catalyst was obtained by dividing the number of total acidic/basic sites by the surface area. Meanwhile, the strength of the acidic/basic sites was denoted by the desorption temperature. For ZrO_2_, the desorption peaks below 250 °C could be attributed to weak acidic/basic sites, the adsorption peaks between 250 °C and 500 °C corresponded to acid/basic sites of medium strength, and the adsorption peaks over 500 °C represented strong acidic/basic sites^56^. Overall, it was proven that the surfactant-assisted nanoparticles increased the number and the density of the total acidic/basic sites compared to bare ZrO_2_. According to the NH_3_-TPD profile (Fig. 4), bare ZrO_2_ showed a small desorption peak at 489 °C, indicating medium acid strength. Regarding the surfactant-assisted nanoparticles, ZrO_2(P123)_ exhibited a small desorption peak at 254 °C, and ZrO_2(CTAB)_ showed a broader desorption peak centered at 266 °C, also indicating medium acidic strength. ZrO_2(CTAB)_ exhibited higher total acidic sites compared to ZrO_2(P123)_ because of the higher surface area of the catalyst^39^. Interestingly, the curves of Bi_2_O_3_/ZrO_2(P123)_ and Bi_2_O_3_/ZrO_2(CTAB)_ with higher total acidic sites compared to their parents ZrO_2_ were shifted to the right. This trend was in general agreement with other findings on Bi_2_O_3_-modified La_2_O_3_ catalysts^7^. Nizah *et al*. found that the addition of Bi_2_O_3_ on the surface of La_2_O_3_ enhanced the acidic properties of the final catalysts^7^. On the basis of the CO_2_-TPD profile (Fig. 5), all catalysts, except for bare ZrO_2_, exhibited basic sites of weak strength at a desorption temperature between 112 °C and 118 °C. Instead, bare ZrO_2_ presented a small desorption peak at 487 °C, indicating medium basic strength. Apart from the weak strength basic sites, ZrO_2(CTAB)_ displayed multiple desorption peaks at desorption temperatures between 468 °C and 525 °C, indicating medium to strong strength of the basic sites.

**Table 3:** Acidic and basic properties of the synthesized catalysts.

Catalyst	Total acidic site (mmol/g)	Density of total acidic site (mmol/m^2^)	Total basic site (mmol/g)	Density of total basic site (mmol/m^2^)
ZrO_2_	0.08	0.002	0.17	0.004
ZrO_2(P123)_	0.35	0.004	1.66	0.021
Bi_2_O_3_/ZrO_2(P123)_	0.41	0.006	1.49	0.024
ZrO_2(CTAB)_	16.12	0.055	6.68	0.022
Bi_2_O_3_/ZrO_2(CTAB)_	17.38	0.111	4.36	0.027

**Figure 6:**
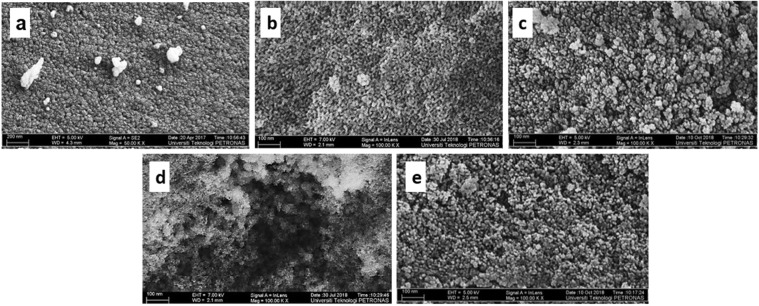
Field electron scanning electron microscope (FESEM) images of (**a**) bare ZrO_2_, (**b**) ZrO_2(P123)_, (**c**) Bi_2_O_3_/ZrO_2(P123)_, (**d**) ZrO_2(CTAB)_, and (**e**) Bi_2_O_3_/ZrO_2(CTAB)_ catalysts.

The morphologies of the synthesized catalysts are illustrated in Fig. 6(a–e). Figure 6a depicts the small pore openings of bare ZrO_2_, and Fig. 6b shows the spherical nanoparticles of ZrO_2(P123)_ (10–20 nm) with ordered arrangement. ZrO_2(CTAB)_ exhibited a rough catalyst surface, and irregular shapes of particles with large external pores were observed between the particles (Fig. 6d). In this analysis, the effects of the surfactant’s hydrophobic tail length were clearly determined as the long chain length of Pluronic P123 provided a better steric effect than CTAB and allowed the self-organization while significantly preventing the collapse of the pore network during the drying process^54^. In addition, the uniform size and arrangement of the ZrO_2(P123)_ particles explained the narrower pore size distribution plot of ZrO_2(P123)_ compared to ZrO_2(CTAB)_. As shown in Fig. 6c,e, the deposition of Bi_2_O_3_ on the outer surface of the catalysts resulted in agglomeration of the Bi_2_O_3_/ZrO_2(P123)_ and Bi_2_O_3_/ZrO_2(CTAB)_ morphologies, respectively. This observation agreed with the large nanoparticle and crystallite sizes obtained previously for Bi_2_O_3_/ZrO_2(P123)_ and Bi_2_O_3_/ZrO_2(CTAB)_. Supplementary Figs. S2(a,b) depicts the energy-dispersive X-ray (EDX) and mapping analyses that were applied to measure the elemental composition and distribution for Bi_2_O_3_/ZrO_2(P123)_ and Bi_2_O_3_/ZrO_2(CTAB)_, respectively. On the basis of the EDX spectrum, three distinct phases of Zr, Bi, and O were clearly observed, which confirmed the presence of Bi_2_O_3_ on the surface of the ZrO_2_ catalyst. The concentrations of Bi in Bi_2_O_3_/ZrO_2(P123)_ and Bi_2_O_3_/ZrO_2(CTAB)_ were also in agreement with the amount loaded during the preparation of the catalysts. In addition, the results of the mapping analysis showed that the Bi_2_O_3_ particles were evenly dispersed on the surface of ZrO_2_ owing to the homogeneous structure of the Bi_2_O_3_/ZrO_2(P123)_ and Bi_2_O_3_/ZrO_2(CTAB)_ catalysts.

### Catalytic activity toward biodiesel production from microalgae

**Figure 7:**
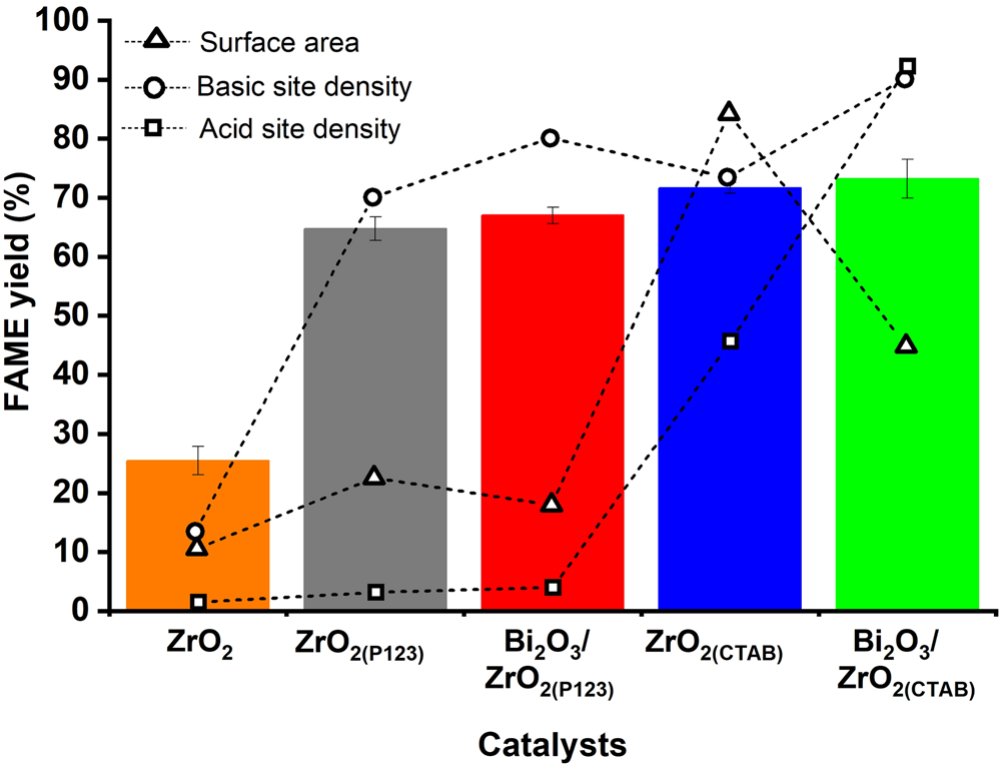
Catalytic activity of the synthesized catalysts for biodiesel production from *Nannochloropsis* sp. lipid.

The simultaneous esterification–transesterification of *Nannochloropsis* sp. lipid to biodiesel was selected as the model reaction to test the activity of the synthesized catalysts. The catalytic activity was evaluated based on the fatty acid methyl esters (FAME) yield, as shown in Fig. 7. In particular, Bi_2_O_3_/ZrO_2(CTAB)_ afforded the highest FAME yield (73.21%), followed by ZrO_2(CTAB)_ (71.65%), Bi_2_O_3_/ZrO_2(P123)_ (67.01%), ZrO_2(P123)_ (64.73%), and bare ZrO_2_ (25.48%). Although the general increase in the surface area of the catalyst by the surfactant-assisted nanoparticles resulted in better FAME yield compared to bare ZrO_2_, the single high surface area did not lead to high catalytic activity. It was found that the catalytic performance is as a result of the synergistic role of both the total acidic and basic site densities. Apart from the lowest surface area and acidic/basic site densities, the least FAME yield obtained by bare ZrO_2_ was also correlated with the small pore openings of the catalyst structure, which prevented the bulky triglyceride molecules from reaching the catalyst’s active site. Similar findings have been observed in other studies. Omar *et al*. found that the balanced acidity and basicity on the surface of Sr/ZrO_2_ catalysts contributed to high FAME yields from waste cooking oil^19^. In another study, a bifunctional catalyst of Bi_2_O_3_-modified La_2_O_3_ was employed for simultaneous esterification–transesterification of Jatropha oil to biodiesel^7^. The results of the current study also showed that the performance of the catalyst was associated with the high surface area and the strong acidic and basic sites, and the mixed oxide catalysts exhibited higher catalytic performance than their parent ZrO_2_. Similarly, Umdu *et al*. investigated the use of single metal oxides and mixed oxides toward the production of biodiesel from microalgae lipid^17^. It was demonstrated that pure CaO and MgO were inactive, but mixed oxides of CaO/Al_2_O_3_ and MgO/Al_2_O_3_ were catalytically active for transesterification under the same reaction conditions. Their high catalytic performance was attributed to the high density and the mild strength of their basic sites.

### Surfactant-assisted sol-gel method followed by hydrothermal treatment

**Figure 8:**
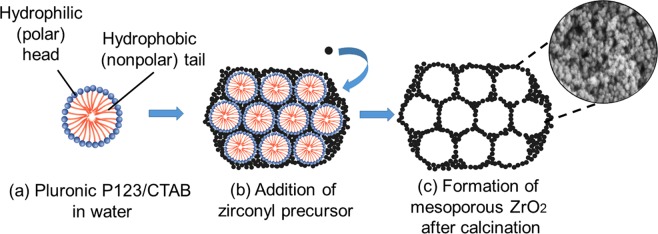
Possible Pluronic P123 and CTAB templating routes for the formation of ZrO_2_ catalysts.

There are three main reactions involved in the sol-gel process, namely, hydrolysis, condensation, and aging^43^. During hydrolysis, H_2_O is replaced by an OH group because of the loss of protons. The condensation reaction leads to the construction of M–OH–M (ol) or M–O–M (oxo) bridges after the elimination of the water molecules^57^. The aging process makes the gel more resistant to capillary stress and increases its mechanical properties^57^. Ward and Ko reported two main concepts of the sol-gel process^58^. In the first concept, a gel is formed because of the condensation of partially hydrolyzed species into a three-dimensional polymeric network. And in the second concept, the properties of the gel depend significantly on the synthesis conditions. The surfactants used in the current study played a decisive role in the development of the catalysts by generating a good porous structure that contributed to the high specific surface area^2^. Figure 8 shows the possible Pluronic P123 and CTAB templating routes for the formation of ZrO_2_ nanoparticles. Both CTAB and Pluronic P123 are well dispersed in polar solvents (especially water) to form micelles, which consist of a hydrophilic head (outward arrangement) and a hydrophobic tail (Fig. 8a). When added into the template solution, the zirconyl precursor assembles and attaches to the hydrophilic head, as shown in Fig. 8b. The zirconyl precursor solution is naturally acidic (pH <1). Under acidic conditions, hydrolysis occurs at a faster rate than condensation and results in a weak branched gel^59^. The addition of ammonia increases the pH of the solution. At this stage, the condensation accelerates compared to hydrolysis, thus forming a rigid gel. Upon stirring, the zirconyl precursor spreads uniformly in the template solution. Internal pressure is generated when the gel is transferred in the autoclave, where the employed heat treatment forces out the micelles and prevents the aggregation of the zirconyl particles, thus contributing to a high surface area^7^. Finally, removal of the template at 500 °C provides a large number of pores with a high pore volume of ZrO_2_ (Fig. 8c).

### Reaction mechanism of biodiesel production using a bifunctional acid–base catalyst

**Figure 9:**
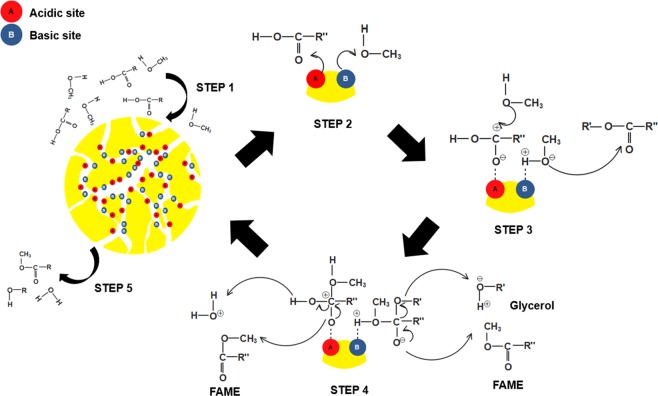
The reaction mechanism of biodiesel production using a bifunctional acid–base catalyst.

In heterogeneous catalysis, the adsorption of the reactants and the desorption of the products occur on the catalyst’s surface. Therefore, both acidic and basic properties of the catalyst are important to achieve a simultaneous esterification and transesterification of high-FFA-content feedstock. Figure 9 shows the possible mechanism for the simultaneous reactions using the bifunctional catalyst. The five steps for a bifunctional catalytic reaction involve (1) diffusion of reactants, (2) physical adsorption of reactants, (3) surface reaction, (4) desorption of products, and (5) diffusion of products. In the first step, the FFA carbonyl group (fatty acid ester) and methanol are diffused from the bulk of solution to the internal catalyst’s surface through catalyst pores. In the second step, the FFA carbonyl group is adsorbed on the acidic site (esterification), and a methanol molecule is adsorbed on the basic site (transesterification) of the catalyst’s surface, thereby affording a carbocation and an oxygen anion, respectively. In the third step, a tetrahedral intermediate is formed via a nucleophilic attack of the alcohol to the esters for both acidic and basic sites. In the fourth step, the hydroxyl group is distracted from the tetrahedral intermediate to form one molecule of water and one molecule of FAME on the acidic site. On the basic site, the C–O bond breaks to form one molecule of FAME and glycerol as by products. The products are desorbed from the catalyst surface as the reaction progresses. In the final step, all the products are diffused from the catalyst’s surface to the bulk of the solution and all the steps were repeated for cleavage of each fatty acid ester^19,60^.

## Materials and Methods

### Materials

Zirconyl nitrate hydrate (ZrO(NO_3_)_2_.xH_2_O, 99%) and bismuth nitrate pentahydrate (Bi(NO_3_)_3_.5H_2_O, 98%) were purchased from Aldrich. The surfactants, namely, poly(ethylene oxide)-block-poly(propylene oxide)-block-poly(ethylene oxide) (Pluronic P123, 90%) and cetyltrimethylammonium bromide (CTAB, 90%), were supplied by Sigma. The aqueous ammonium solution (25%) was purchased from Merck. Marine microalgae of *Nannochloropsis* sp. used as the biodiesel feedstock were supplied by Laboratory & Scientific Enterprise, Malaysia.

### Catalyst preparation

A sol-gel method followed by a hydrothermal treatment was adopted for the synthesis of bare and surfactant-assisted ZrO_2_. On the basis of the typical synthesis method, 8.1 g of ZrO(NO_3_)_2_.xH_2_O was dissolved in 30 mL of distilled water, followed by the addition of 120 mL of absolute ethanol. The solution was vigorously stirred for about 20 min at room temperature to achieve homogenization. An aqueous ammonium solution (25%) was then added dropwise to the above solution until pH 8 was attained. The new solution was continuously stirred until gelling, and the obtained sample was transferred into a Teflon-lined autoclave. The vessel was sealed and heated in an oven at 120 °C for 24 h. The resulting gel was washed several times with distilled water and ethanol, dried at 120 °C, and calcined at 500 °C for 4 h. The surfactant-assisted ZrO_2_ samples were prepared similarly to the bare ZrO_2_, and the surfactant was added after the addition of absolute ethanol. The obtained calcined bare ZrO_2_, Pluronic P123-assisted ZrO_2_, and CTAB-assisted ZrO_2_ samples were designated as ZrO_2_, ZrO_2(P123)_, and ZrO_2(CTAB)_, respectively.

### Optimization study

A four-factor RSMCCD was implemented to study the effects of the independent parameters, i.e., surfactant/Zr molar ratio (A: 0.01–0.05 for Pluronic P123^40^ and 0.6–1.0 for CTAB^39^), pH (B: 9–11), aging time (C: 12–48 h), and temperature (D: 80–120 °C), on the surface area of ZrO_2_. The statistical calculations were performed using Design Expert Version 11 (STAT-EASE Inc., Minneapolis, USA). The experimental values were compared with the predicted values to test the adequacy of the final reduced model. The recommended optimum conditions were experimentally implemented to validate the optimum surface area value predicted by the model.

### Impregnation with Bi_2_O_3_

To investigate the catalytic activities of mixed zirconia oxides, Bi_2_O_3_/ZrO_2_ samples were prepared via a simple incipient wetness impregnation method. The representative ZrO_2_ was selected based on the optimum surface areas of ZrO_2(P123)_ and ZrO_2(CTAB)_. 5.8 g of Bi(NO_3_)_3_·5H_2_O (which corresponded to 5 wt.% of Bi_2_O_3_) was dissolved in distilled water, and 47.5 g of ZrO_2(P123)_ and ZrO_2(CTAB)_ were added (separately) into the metal solution, which was stirred for 24 h. Water was then removed by drying in an oven at 120 °C and subsequently by calcination at 500 °C for 5 h using a muffle furnace. The samples were designated as Bi_2_O_3_/ZrO_2(P123)_ and Bi_2_O_3_/ZrO_2(CTAB)_.

### Catalyst characterization

The structure of the surfactant-assisted ZrO_2_ catalysts was characterized by small-angle XRD analysis (Bruker AXS D8). The scanning was performed with a step of 0.02° in a 2θ range of 0° to 10°. The crystalline phases of the catalyst were characterized using wide-angle powder XRD analysis (PAN Analytical X′pert3 Powder & Empyrean) coupled with Cu-Kα radiation. The scanning was performed with a step of 0.02° and 2 s per step in a 2θ range of 10° to 80°. The crystallite sizes were defined by adopting the Debye–Scherrer formula based on the highest crystal peak. The structure of the crystalline phases was refined using the Rietveld method.

The surface area, total pore volume, and pore size distribution were acquired from nitrogen adsorption–desorption isotherms using an adsorption porosimeter (Micromeritics ASAP 2020) at 78 K. Prior to the measurement, the catalyst was treated in vacuum at 200 °C to remove the moisture adsorbed from the catalyst surface and pores^56^.

The acidic and basic properties of the catalyst were measured by temperature-programmed desorption (TPD; Thermo Scientific TPDRO 1100) of ammonia (NH_3_) and carbon dioxide (CO_2_). During the pre-treatment process, the samples were treated with helium (He) gas for 10 min at a rate of 20 mL/min. Then, the temperature was increased to 150 °C at a rate of 10 °C/min and was kept constant for 45 min. After being cooled down to 50 °C, the pre-treated samples were saturated with NH_3_ or CO_2_ at a rate of 20 mL/min and kept under these conditions for 60 min. Then, the samples were purged with He at a rate of 20 mL/min for 20 min to avoid physisorption and remove the remaining NH_3_ or CO_2_. Finally, the desorption of NH_3_ or CO_2_ was performed under He flow at a rate of 20 mL/min, and the samples were heated up to 700 °C at a rate of 10 °C/min, where they were maintained for 60 min^56^.

The morphology of the catalyst was captured using a FESEM microscope coupled with an EDX spectrometer (Ziess Supra 55VP) operating at 5 kV.

### Biodiesel production and gas chromatography analysis

All of the catalytic reactions for biodiesel production were performed in a 50 mL three-necked flask equipped with a condenser and a stirrer. In this study, constant reaction conditions were employed using a lipid/methanol ratio of 1:90 (g/mL) and a catalyst loading of 20 wt.% at 80 °C for 6 h. The upper layer, containing FAME, was separated from the heterogeneous catalyst by centrifugation. The biodiesel yield was measured using gas chromatography with a flame ionization detector (GC-FID; Shimadzu GC-2010). BPX-20 was used as the column, with He as the carrier gas at a flow rate of 1.73 mL/min and a pressure of 83.9 kPa. The temperature of the column was first set at 150 °C and increased to 240 °C at a rate of 5 °C/min. Both the injector and FID temperatures were set at 250 °C. The biodiesel yield (%) was calculated using Eqs (3) and (4),

3 $${FAME}\,{Content}( \% )=\frac{{\Sigma A}_{FAME}-{A}_{ISTD}}{{A}_{ISTD}}\times \frac{{C}_{ISTD}\times {V}_{ISTD}}{m}\times 100$$

Where, ∑A_FAME_ is the total peak area of FAME, A_ISTD_ is the peak area of the internal standard, C_ISTD_ is the concentration of the internal standard (mg/mL), V_ISTD_ is the volume of the internal standard (mL), and m is the sample mass (mg).

4 $${FAME}\,{Yield}( \% )={FAME}\,{content}\,{from}\,GC\times \frac{{weight}\,{of}\,{biodiesel}}{{weight}\,{of}\,{microalgae}\,{lipid}}\times 100$$

## Conclusion

RSMCCD was successfully employed to investigate the effect of various parameters on the surfactant-enhanced surface area of ZrO_2_. The high R^2^ obtained for ZrO_2(P123)_ and ZrO_2(CTAB)_ indicated that the empirical models derived from RSMCCD can effectively describe the relationship between the process parameters and the response (ZrO_2_ surface area). The physicochemical analyses revealed that ZrO_2(P123)_ and ZrO_2(CTAB)_ possess better surface area, pore structure, and acidic and basic properties compared to bare ZrO_2_. Moreover, the addition of Bi_2_O_3_ on ZrO_2_ improved the density of total acidic and basic sites of Bi_2_O_3_/ZrO_2(P123)_ and Bi_2_O_3_/ZrO_2(CTAB)_. Nevertheless, the success of the catalytic activity on simultaneous esterification and transesterification of microalgae lipid to biodiesel does not directly depend on the high surface area of the catalysts. In fact, the high density of the total acidic and basic sites is required for a high FAME yield. Thus, the development of bifunctional Bi_2_O_3_/ZrO_2_ catalysts has a high chance of simplifying the biodiesel production process using low-grade high-FFA feedstock.

## Supplementary Information

Supplemental Information
