# Supplementary material for: Tailoring the surface area and the acid–base properties of ZrO2 for biodiesel production from Nannochloropsis sp
Source: Sci Rep. 2019 Nov 7;9:16223. doi: 10.1038/s41598-019-52771-9 (PMC6838316; doi:10.1038/s41598-019-52771-9)
Supplement: Supplementary file 1 — Supplemental Information [file 41598_2019_52771_MOESM1_ESM.pdf]

Tailoring the surface area and the acid–base properties of ZrO_2 for biodiesel production from *Nannochloropsis* sp.

Nurul Jannah Abd Rahman[1], Anita Ramli[1], Khairulazhar Jumbri[1,+], and Yoshimitsu Uemura[2,+]

Run order	A: Surfactant/Zr	B: pH	C: Aging time (h)	D: Temperature (°C)	Surface area (m^2/g)	
					Predicted	Actual
1	0.02	10.5	21	110	77	78
2	0.03	10.0	30	100	64	63
3	0.03	10.0	30	100	64	65
4	0.02	9.5	39	110	65	66
5	0.02	10.5	39	110	66	65
6	0.03	10.0	30	100	64	63
7	0.02	9.5	21	110	77	77
8	0.03	10.0	30	100	64	62
9	0.03	11.0	30	100	67	66
10	0.02	9.5	39	90	67	66
11	0.03	10.0	30	80	64	65
12	0.04	9.5	39	110	69	69
13	0.03	10.0	30	100	64	66
14	0.02	10.5	39	90	69	69
15	0.02	9.5	21	90	63	64
16	0.05	10.0	30	100	64	64
17	0.04	10.5	39	90	70	70
18	0.04	9.5	39	90	68	67
19	0.04	10.5	21	110	78	78
20	0.04	9.5	21	90	67	67
21	0.04	9.5	21	110	78	79
22	0.03	10.0	48	100	71	72
23	0.03	9.0	30	100	77	76
24	0.03	10.0	30	120	76	75
25	0.03	10.0	12	100	78	77
26	0.04	10.5	21	90	68	68
27	0.03	10.0	30	100	64	66
28	0.02	10.5	21	90	64	64
29	0.01	10.0	30	100	60	59
30	0.04	10.5	39	110	70	70

Supplementary Table S1. Reaction conditions and surface area of ZrO_2 using the Pluronic P123-assisted approach.

[1]Fundamental and Applied Sciences Department, Universiti Teknologi PETRONAS, 32610 Seri Iskandar, Perak, Malaysia. [2]Centre for Biofuel and Biochemical Research, Universiti Teknologi PETRONAS, 32610 Seri Iskandar, Perak, Malaysia. Correspondence and requests for materials should be addressed to A.R. (email:anita_ramli@utp.edu.my). [+]These authors contributed equally to this work.

Run order	A: Surfactant/Zr	B: pH	C: Aging time (h)	D: Temperature (°C)	Surface area (m^2/g)	
					Predicted	Actual
1	0.90	10.5	39	110	228	224
2	0.80	10.0	30	100	274	294
3	0.80	10.0	30	100	274	250
4	0.80	10.0	30	100	274	276
5	0.80	10.0	30	80	150	143
6	0.80	10.0	12	100	64	57
7	0.70	9.5	21	90	53	58
8	0.60	10.0	30	100	61	58
9	0.80	10.0	30	100	274	278
10	0.90	9.5	39	90	56	61
11	0.70	10.5	39	90	63	61
12	0.90	10.5	39	90	58	63
13	0.80	10.0	30	120	180	173
14	0.70	10.5	39	110	209	215
15	0.70	9.5	39	110	258	257
16	0.90	9.5	21	90	54	61
17	0.90	10.5	21	90	56	58
18	0.70	10.5	21	110	61	58
19	0.80	10.0	30	100	274	270
20	0.90	9.5	39	110	282	295
21	0.70	10.5	21	90	60	61
22	0.80	11.0	30	100	58	59
23	0.80	9.0	30	100	72	56
24	0.80	10.0	48	100	168	160
25	0.90	9.5	21	110	48	51
26	0.70	9.5	21	110	45	53
27	0.70	9.5	39	90	57	64
28	0.90	10.5	21	110	59	65
29	0.80	10.0	30	100	274	278
30	1.00	10.0	30	100	70	59

Supplementary Table S2. Reaction conditions and surface area of ZrO_2 using the CTAB-assisted approach.

Source	Sum of squares	DF	Mean square	F-value	P-value
Model	844.47	16	52.78	33.43	<0.0001
A-Surfactant/Zr	33.11	1	33.11	20.97	0.0005
B-pH	50.50	1	50.50	31.99	<0.0001
C-Aging time	77.22	1	77.22	48.91	<0.0001
D-Temperature	179.58	1	179.58	113.74	<0.0001
AB	0.0023	1	0.0023	0.0014	0.9704
AC	0.0005	1	0.0005	0.0003	0.9860
AD	0.0189	1	0.0189	0.0120	0.9145
BC	1.23	1	1.23	0.7768	0.3941
BD	3.29	1	3.29	2.08	0.1728
CD	162.24	1	162.24	102.76	<0.0001
A^2	6.93	1	6.93	4.39	0.0563
B^2	97.19	1	97.19	61.56	<0.0001
C^2	194.61	1	194.61	123.25	<0.0001
D^2	65.68	1	65.68	41.60	<0.0001
ACD	7.74	1	7.74	4.90	0.0453
A^2B	49.23	1	49.23	31.18	<0.0001
Residual	20.53	13	1.58		
Lack of Fit	8.39	8	1.05	0.4323	0.8603
Pure Error	12.13	5	2.43		
Cor Total	864.99	29			
Std. dev. = 1.26; CV% = 1.83; R^2 = 0.9763; Adjusted R^2 = 0.9471; Predicted R^2 = 0.8667					

Supplementary Table S3. Relationship between the independent parameters and the surface area of the reduced cubic model of $ZrO_{2(P123)}$ as determined by the ANOVA test.

Source	Sum of squares	DF	Mean square	F-value	P-value
Model	2.707E+05	18	15039.58	76.05	<0.0001
A-Surfactant/Zr	118.84	1	118.84	0.6009	0.4546
B-pH	324.22	1	324.22	1.64	0.2267
C-Aging time	5335.95	1	5335.95	26.98	0.0003
D-Temperature	454.48	1	454.48	2.30	0.1577
AB	24.17	1	24.17	0.1222	0.7332
AC	98.36	1	98.36	0.4974	0.4953
AD	177.44	1	177.44	0.8973	0.3639
BC	1088.64	1	1088.64	5.51	0.0387
BD	556.81	1	556.81	2.82	0.1215
CD	35622.40	1	35622.40	180.14	<0.0001
A^2	74514.19	1	74514.19	376.80	<0.0001
B^2	75203.86	1	75203.86	380.29	<0.0001
C^2	42991.44	1	42991.44	217.40	<0.0001
D^2	20484.00	1	20484.00	103.58	<0.0001
ACD	121.72	1	121.72	0.6155	0.4493
BCD	1061.51	1	1061.51	5.37	0.0408
A^2C	2735.32	1	2735.32	13.83	0.0034
A^2D	7768.57	1	7768.57	39.28	<0.0001
Residual	2175.29	11	197.75		
Lack of Fit	1166.76	6	194.46	0.9641	0.5268
Pure Error	1008.53	5	201.71		
Cor Total	2.729E+05	29			
Std. dev. = 14.06; CV% = 10.24; R^2 = 0.9920; Adjusted R^2 = 0.9790; Predicted R^2 = 0.8388					

Supplementary Table S4. Relationship between the independent parameters and the surface area of the reduced cubic model of $ZrO_{2(CTAB)}$ as determined by the ANOVA test.

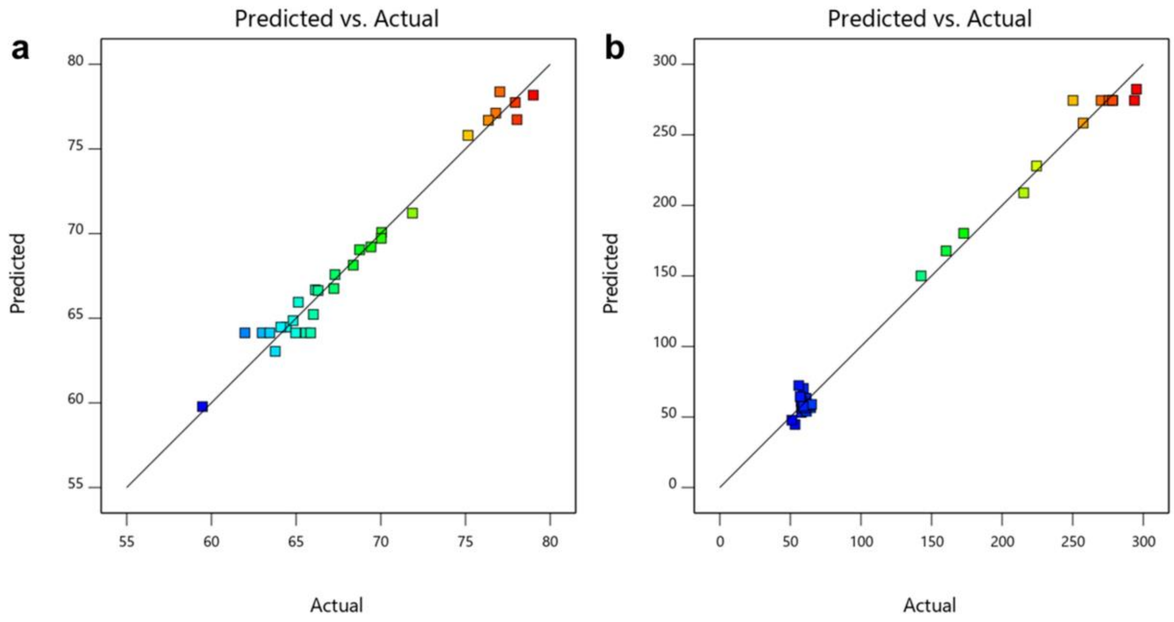

Supplementary Fig. S1. Correlation between predicted and actual values of the surface areas of (**a**) $ZrO_{2(P123)}$ and (**b**) $ZrO_{2(CTAB)}$.

Catalyst	Preparation conditions				Predicted surface area (m^2/g)	Actual surface area (m^2/g)	Deviation (%)
	A	B	C	D			
$ZrO_{2(P123)}$	0.03	9.50	22	110	80	79	1.25
$ZrO_{2(CTAB)}$	0.89	9.80	39	110	304	295	2.96

Supplementary Table S5. Optimum reaction conditions for the synthesis of $ZrO_{2(P123)}$ and $ZrO_{2(CTAB)}$ catalysts with high surface area.

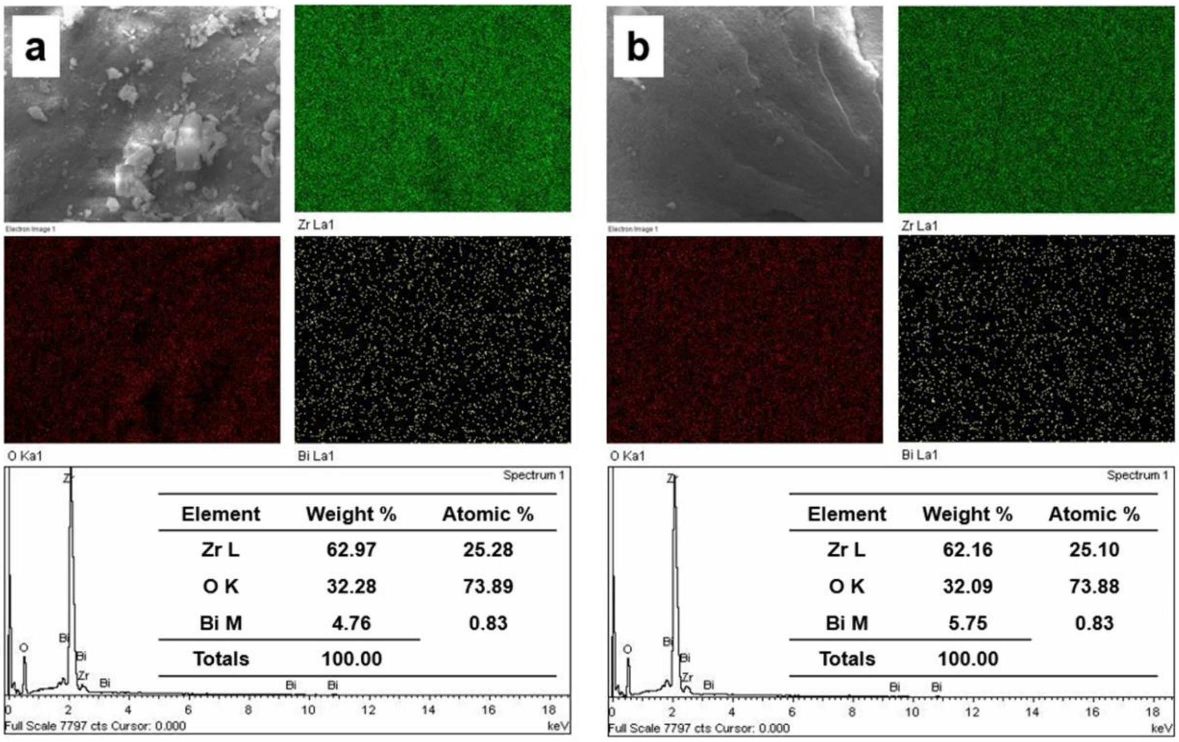

Supplementary Fig. S2. The mapping and EDX analyses of the (**a**) $Bi_2O_3/ZrO_{2(P123)}$ and (**b**) $Bi_2O_3/ZrO_{2(CTAB)}$ catalysts.
